# Pediatric Cardio-Oncology: From Gap in Evidence to Future Perspectives

**DOI:** 10.3390/diagnostics16020268

**Published:** 2026-01-14

**Authors:** Adriana Correra, Valeria Cetoretta, Anna Chiara Maratea, Serena Ferrara, Isabella Di Sarno, Vincenzo Russo, Federico Guerra, Alfredo Mauriello, Antonello D’Andrea

**Affiliations:** 1 Cardiology Department, Ospedali Riuniti University Hospital, Viale Pinto 1, 71122 Foggia, Italy; adrianacorrera@gmail.com; 2 Cardiology and Arrhythmology Clinic, University Hospital “Ospedali Riuniti”, Marche Polytechnic University, Via Conca 71, 60126 Ancona, Italy; vcetoretta@gmail.com (V.C.); guerra.fede@gmail.com (F.G.); 3 Department of Cardiovascular Disease, ASL Napoli 1 Centro, Via Comunale del Principe, 13/a, 80145 Napoli, Italy; annachiara.maratea@gmail.com; 4 Pediatric Department, ASL Napoli 2 Nord, Via Padre Mario Vergara, 228, Frattamaggiore, 80027 Naples, Italy; serena.ferrara@hotmail.it; 5 UOSD Neurophysiopathology, AORN “Santobono Pausillipon”, Via Mario Fiore, 6, 80129 Naples, Italy; isadisarno@gmail.com; 6 Cardiology Unit, Department of Medical and Translational Sciences, University of Campania “Luigi Vanvitelli”, “V. Monaldi” Hospital, Via Leonardo Bianchi snc, 80131 Naples, Italy; vincenzo.russo@unicampania.it; 7 S.C. Cardiology, Institute National Cancer, IRCCS, Fondazione “G. Pascale”, Via M. Semmola 52, 80131 Naples, Italy; 8 Cardiology and Intensive Care Unit, Department of Cardiology, “Umberto I” Hospital, Via Alfonso De Nicola 1, 84014 Nocera Inferiore, Italy; antonellodandrea@libero.it

**Keywords:** cardio-oncology, cardiotoxicity, pediatric health, cancer

## Abstract

Improved survival rates for paediatric cancer patients represent a major medical achievement, but they have simultaneously brought the long-term sequelae of oncological treatments into sharp focus. Cardiotoxicity stands out as one of the most serious complications, being the leading cause of non-relapse-related morbidity and mortality among childhood cancer survivors. This comprehensive review analyses the current landscape, highlighting the significant gap in evidence that hinders optimal care. This paper constitutes a comprehensive narrative and scoping review based on a critical analysis of current clinical guidelines, landmark studies, and consensus papers in paediatric cardio-oncology. Crucially, it assesses the heterogeneity and limitations of existing evidence regarding standardized surveillance protocols, primary prevention strategies, and acute/late-onset cardiovascular complication management. The review then identifies and critically discusses key areas for future research and clinical development. A critical gap in evidence persists in paediatric cardio-oncology, leading to significant variability in clinical practice and the underdiagnosis/undertreatment of cardiovascular risk factors in this vulnerable population. To bridge this gap, there is an urgent need for international collaborative research. The overarching goal is to transform paediatric cardio-oncology into a predictive and preventive speciality, ensuring that all childhood cancer survivors achieve not only extended life expectancy but also improved cardiovascular quality of life.

## 1. Introduction

The field of paediatric oncology has achieved a historic triumph with five-year survival rates now surpassing 80% [1]. This dramatic improvement is a testament to decades of progress, fuelled by advancements in chemotherapy, refined supportive care, and the introduction of groundbreaking therapeutics like chimeric antigen receptor T-cell therapy (CAR-T) and immune checkpoint inhibitors (ICI) [1].

However, this success has unveiled a critical, new challenge. As more children survive cancer, they face a significantly elevated risk of long-term health issues, specifically cardiovascular disease. Survivors experience a five- to six-fold increase in cardiovascular disease risk, making it the leading non-cancer cause of death in this population [1,2].

The spectrum of this cardiotoxicity is broad, encompassing severe conditions such as symptomatic and asymptomatic heart failure, arrhythmias, coronary artery disease, valvular disease, pericardial disease, hypertension, and peripheral vascular disease [3,4].

A significant concern is that many patients remain asymptomatic for extended periods. Without appropriate early screening, they may not seek care until the disease is advanced, underscoring the vital need for vigilant, long-term cardiovascular monitoring [5].

This comprehensive review analyses the current landscape, highlighting a significant evidence gap that hinders optimal care.

## 2. Materials and Methods

This study was conducted as a clinically oriented scoping comprehensive review, with the aim of mapping the current evidence in paediatric cardio-oncology, identifying key knowledge gaps, and providing a pragmatic synthesis to support clinical decision-making, rather than performing a formal systematic review or meta-analysis.

A structured literature search was conducted across PubMed/MEDLINE and EMBASE, including articles published between January 2010 and December 2025. The search strategy combined terms related to paediatric populations, cardio-oncology and cardiotoxicity, childhood malignancies (including leukaemia, lymphoma, brain tumours, sarcomas, neuroblastoma, and other solid tumours), and anticancer treatments associated with cardiovascular toxicity, encompassing conventional chemotherapy (anthracyclines and alkylating agents), targeted therapies (ABL, ALK, BRAF/MEK, multi-kinase and mTOR inhibitors), immunotherapies (bispecific antibodies, CAR-T cells, immune checkpoint inhibitors, anti-CD20 and anti-GD2 antibodies), and radiotherapy.

Eligible sources included clinical studies, prospective and retrospective cohorts, randomized controlled trials, meta-analyses, and registry-based reports addressing cardiovascular toxicity in paediatric oncology patients. Given the heterogeneity of study designs and outcomes, no quantitative synthesis was undertaken. Instead, evidence was synthesized qualitatively, with consideration of study populations, outcome definitions, follow-up duration, and methodological rigor, guided by Joanna Briggs Institute appraisal tools and, where appropriate, the Cochrane Risk-of-Bias framework. Consistent with scoping review methodology, this approach was chosen to ensure breadth of coverage and transparency, acknowledging that a PRISMA-based systematic review was beyond the scope and objectives of the present work.

## 3. Paediatric Cardio-Oncology Landscape

Paediatric cancers are a heterogeneous group of neoplasms that differ profoundly from typical adult forms in terms of biology, response to treatment and prognosis. Globally, their distribution is relatively stable: leukaemia, particularly acute lymphoblastic leukaemia (ALL), accounts for approximately 30% of all diagnoses [6]; followed by brain and central nervous system tumours (≈20–25%) [7], lymphomas (≈10%), neuroblastoma (≈6%), Wilms’ tumour (≈5%), and bone or soft tissue sarcomas (≈10%). In addition to these major categories, there is a diverse set of rare tumours, including liver, germ cell, and other complex solid tumours, which together account for an additional 15–20% of paediatric diagnoses [8].

The latest global data from solid tumour registries alone show approximately 211,000 new cases per year of tumours in children aged 0–14 years in 2022. Including adolescents and areas that are not well registered, estimates rise to ≈300,000–400,000 new cases per year in children aged 0–19 years [9,10]. Current 5-year survival rates now exceed 80%, resulting in a rapidly growing population of long-term childhood cancer survivors who are expected to live for several decades after completion of therapy [1].

Based on real-world cohorts, it is estimated that approximately 60–70% of paediatric cancer patients receive only standard chemotherapy (±radiotherapy) without being exposed to innovative drugs. However, in recent years, the proportion of patients receiving targeted therapies or immunotherapies as part of their treatment regimen has increased. Analyses from large international paediatric cardio-oncology centres show that approximately 25–35% of patients receive at least one targeted or immunotherapy treatment during their disease [11]. The current and prospective outlook suggests that the percentage of children exposed to at least one targeted or immunotherapy treatment is set to increase over the next 5–10 years. Recent international studies indicate that, with the early introduction of BRAF/MEK inhibitors in paediatric gliomas, the expansion of ALK inhibitors in neuroblastoma and the growing integration of bispecific in high-risk ALL, the overall proportion of patients exposed to at least one “innovative” treatment could approach 40–50% in the next decade. However, as highlighted by AHA 2025, the JACC 2022 Consensus, and numerous longitudinal cohorts of survivors, most of the burden of cardiotoxicity in the long-term population will remain dominated by the most common exposures: anthracyclines, high-dose alkylating agents, and radiotherapy to critical areas such as the mediastinum, neck, and skull [12,13,14].

At present, the primary reference for cardiovascular toxicity in pediatric onco-hematology is the Delphi consensus by Toro et al., which provides therapy-oriented recommendations tailored to children and teenagers [15]. However, the authors acknowledge that pediatric data for many targeted and immunotherapies remain scarce, rendering several recommendations expert-based rather than evidence-based. Consequently, substantial variability persists among international experts regarding the timing and frequency of cardiac surveillance and the management of borderline or subclinical abnormalities, including mildly reduced LVEF, abnormal GLS, or isolated diastolic dysfunction—areas that remain true clinical grey zones [16]. As a result, many decisions are still extrapolated from adult cardio-oncology guidelines [17]. In long-term survivors, uncertainty regarding the role of biomarkers and CMR in asymptomatic follow-up further contributes to heterogeneity in real-world practice.

## 4. Cardiovascular Toxicity of Paediatric Cancer Treatments

### 4.1. Cardiovascular Toxicity from Chemotherapy Drugs

Anthracyclines form the backbone of numerous paediatric protocols for acute lymphoblastic and myeloid leukaemia (ALL, AML), Hodgkin’s and non-Hodgkin’s lymphoma, bone and soft tissue sarcomas, neuroblastoma and other solid tumours. Anthracyclines are the primary determinant of dilated or hypotrophic cardiomyopathy, systolic and diastolic left ventricular dysfunction, with a clear dose–response relationship and a risk of heart failure that persists and amplifies decades after exposure [14]. Cohort studies of 14,358 adult survivors of paediatric tumours show a hazard ratio >5 for heart failure, coronary artery disease and valvular heart disease compared to unexposed siblings, confirming the impact of anthracyclines on the burden of late cardiotoxicity 95% (95% Confidence Interval [CI] 3.4–9.6; *p* < 0.001) [18]. In patients still in their developmental years, dysfunction may initially present as hypotrophic remodelling with reduced ventricular mass and thinned walls, often in the absence of clinical symptoms, as documented by Slieker et al. in a multicentre cohort of 546 paediatric survivors: LVEF was reduced in only a minority, while subtle alterations in global longitudinal strain were more frequent, although in most cases remaining within ranges considered “normal” (0.73% [95% CI, 0.32–1.14%]; *p* = 0.001) [19].

Alkylating agents, widely used in regimens for leukaemia, lymphomas, sarcomas, and high-dose protocols with transplantation, are mainly associated with endothelial and vascular toxicity, acute myocardial dysfunction (particularly with high doses of cyclophosphamide), and the onset of cardiometabolic risk factors that add to the direct anthracycline-mediated risk. Analysis derived from the Childhood Cancer Survivor Study from Mansoor et al. confirm that combined exposure to anthracyclines and thoracic RT, often against a background of alkylating chemotherapy, is associated with a cumulative incidence of cardiovascular complications close to 20% at 30 years after diagnosis (odds ratio 3.7, 95% CI 3.2–4.2) [20].

### 4.2. Cardiovascular Toxicity from Radiotherapy

Thoracic and mediastinal radiotherapy (RT) is a pillar of treatment for various paediatric cancers, particularly lymphomas, mediastinal tumours, certain sarcomas and high-risk solid tumours. RT involving the heart is associated with an apparent increase in the risk of ischaemic heart disease, left ventricular dysfunction, valvular disease, especially aortic/mitral insufficiency and stenosis, and pericardial disease, with a risk that increases with the average cardiac dose and can manifest 10–20 years after the end of treatment [14]. A recent review in the field of radiotherapy quantified the risk of coronary events as a function of the average dose to the heart, highlighting that even modest increases in the average cardiac dose are associated with a significant increase in ischaemic events in 24,214 survivor populations (*p* < 0.001) [21]. In children, where RT is often administered to tissues that are still growing, the consequences include not only structural heart disease and coronary artery disease, but also alterations in cardiac growth and potential synergy with other risk factors (e.g., endocrinopathies from cranial RT). The combination of thoracic RT and anthracyclines is particularly harmful: cohort data reported in the AHA statement and in the work of Armstrong et al. [15] show that combined exposure carries an exponentially higher risk of heart failure (*p* = 0.018), valvular (*p* = 0.014) and pericardial disease (*p* = 0.003) than individual therapeutic modalities.

### 4.3. Cardiovascular Toxicity from Targeted Therapies

Targeted therapies include a diverse range of drugs as tyrosine kinase inhibitors (TKIs), BRAF/MEK inhibitors, mTOR inhibitors, and monoclonal antibodies directed against specific antigens. Anti-ABL and multi-kinase TKIs (e.g., imatinib, dasatinib, sunitinib, sorafenib) are associated with prolonged QTc, hypertension, heart failure, and endothelial toxicity; VEGF/multi-kinase inhibitors present a significant risk of hypertension, left ventricular dysfunction, and arterial/venous thrombotic events [13]. BRAF/MEK inhibitors (e.g., dabrafenib and trametinib) are increasingly used in BRAF-mutated paediatric gliomas; available data indicate a risk of reduced ejection fraction and left ventricular dysfunction, mostly mild and reversible, requiring regular monitoring with echocardiography and strain imaging, as discussed in recent reviews on advanced imaging in paediatric survivors. mTOR inhibitors (everolimus, sirolimus) mainly cause metabolic toxicity (dyslipidaemia, hypertension, insulin resistance) that contributes to long-term overall cardiovascular risk, although they are not classically cardiotoxic in the “anthracycline-like” sense [14].

### 4.4. Cardiovascular Toxicity from Immunotherapies

CAR-T cell therapies and immune checkpoint inhibitors (ICIs) represent the cutting edge of paediatric oncology, but they introduce new and often dramatic cardiovascular toxicity profiles. Anti-CD19 CAR-T cells are now approved for refractory or multiple relapse paediatric and young adult B-ALL, and their cardiotoxicity is closely linked to cytokine release syndrome (CRS): hypotension, shock, tachycardia, arrhythmias, acute left ventricular dysfunction and, in the most severe cases, cardiac arrest [14].

Observational studies and reviews on heart transplantation in survivors show that a minority of patients may progress to terminal heart failure refractory to medical therapy, making heart transplantation a viable option, with outcomes comparable to other forms of dilated cardiomyopathy (*p* = not significant) [22].

ICIs, used in selected subgroups of relapsed/refractory Hodgkin’s lymphoma and in some MSI-H/dMMR or TMB-high solid tumours, have been associated in adults with immune myocarditis, sometimes fulminant, as well as pericarditis, arrhythmias and forms of non-ischaemic cardiomyopathy. Although adverse events are rare in children, their potential lethality requires a high level of attention, early recognition and aggressive immunosuppressive treatment [13].

Bispecific antibodies are an important advance in pediatric leukemia, particularly in relapsed/refractory B-ALL or MRD-positive disease. Cardiovascular toxicity is mainly functional and inflammation-related, with cytokine release syndrome—generally milder than with CAR-T—causing tachycardia, hypotension, and transient hemodynamic instability. Direct cardiotoxicity is rare, but risk increases in patients with prior anthracycline exposure or pre-existing myocardial vulnerability [23].

Anti-cluster of differentiation (CD20) and anti-disialoganglioside (GD)2 antibodies are key components of consolidation therapy in high-risk neuroblastoma, significantly improving survival when combined with growth factors and retinoids. They generally have a favourable cardiac profile but may cause hypotension, tachycardia, vasospasm and, in the case of anti-GD2 antibodies, catecholaminergic storms during infusion with acute haemodynamic effects. This response may increase myocardial oxygen demand, leading to transient ischaemia and occasional arrhythmias. Structural cardiac events are rare, but intensive monitoring during infusion is required [24].

Overall, therefore, while anthracyclines, alkylating agents and radiotherapy account for the bulk of the burden of chronic cardiotoxicity in long-term survivors, targeted and immunotherapeutic therapies introduce often severe acute and subacute toxicity phenotypes, affecting a smaller but rapidly expanding proportion of the paediatric cancer population.

The pharmacological classes with their respective therapeutic indications and main cardiovascular toxicities are shown in Table 1.

## 5. Diagnostic Tools for Cardiotoxicity in Children

### 5.1. Open Issues in Transthoracic Echocardiography (TTE) Cardiotoxicity Assessment

Transthoracic echocardiography (TTE) remains the cornerstone of cardiac monitoring in children undergoing cancer treatment and is the tool on which all AHA 2025 recommendations for screening and surveillance of cardiotoxicity in children are based [14,15,16].

The most commonly used indices are the shortening fraction (SF) and left ventricular ejection fraction (LVEF), measured using one-dimensional and biplanar Simpson methods [14]. This works for intensively treated or heavily pre-exposed patients, in whom a clinically relevant decline in systolic function may become apparent, with reported incidence of cancer therapy-related cardiac dysfunction in up to 24% of patients [∆LVEF−3.3% (IQR: −7.8, 0%), p<0.0001] in small case series [12]; however, in unselected pediatric populations, conventional global parameters remain relatively insensitive for the detection of subclinical cardiotoxicity.

Across large CCS cohort exposed to anthracyclines, Slieker et al. found that only 0.8% of patients had an LVEF < 50%, while the majority had global systolic function within the normal range even years after the end of chemotherapy [19]. A prospective study by Mokshagundam et al. in CCS treated with anthracyclines showed that changes in LVEF and FS (e.g., from 65% to 60%) may represent real myocardial damage, even while remaining within an apparently normal range. The authors emphasise that echocardiography alone risks underestimating damage if not supplemented with more sensitive measures and biomarkers (*p* < 0.001) [25].

However, when observing patients who develop cardiomyopathy at a distance, EF and FS show a measurable decline years earlier [11,12]; therefore, they are not useless, but they are slow and require time series. In a small retrospective case–control study conducted on 50 patients and 50 controls, Venturelli et al. confirm normal LVEF and FS data, albeit with statistical differences vs. controls, highlighting significant differences in diastolic indices: IVRT, DT, E/A waves, even without alteration of the E/A ratio in the classic pattern. TDI also shows differences in myocardial velocities (S’, E’, A’) on the septum and posterior wall; E/E’ and E’/A’ do not change significantly in a picture that is not clear in terms of increased filling pressures. Multivariate analysis shows allogeneic bone marrow transplantation (HSCT) to be the factor with the greatest impact on measurements [26]. The tendency to alter diastole early is confirmed by Slieker’s more robust study of 500 patients, which found a longer IVRT in CCS, while E/A and E/E’ often do not differ markedly [19]. Diastolic impairment could therefore be a sign of subclinical cardiotoxicity, but it remains preload/afterload-sensitive and does not always provide clear diagnostic patterns.

Overall, conventional echocardiography is indispensable for serial assessment but is not sufficient for the early identification of patients at higher risk of cardiotoxicity.

### 5.2. Open Issues in Speckle Tracking Echocardiography Cardiotoxicity Assessment

The introduction of speckle tracking echocardiography (STE) has enabled the measurement of myocardial strain parameters (GLS, GCS, segmental strain) in paediatric patients. Cardio-oncology guidelines and consensus documents explicitly recommend the use of GLS in adult patients treated with anthracyclines, but emphasise that paediatric evidence is still evolving [17].

In a multicentric cross-sectional study of 500 patients, Slieker et al. demonstrated that the mean GLS was significantly lower in CCS than in healthy controls; however, only 7.7% had a longitudinal strain value with a Z-score < −2 (defined as abnormal), and the absolute difference in GLS with controls was modest (approximately 0.7%) (*p* < 0.001) [19]. The authors conclude that, in the medium-term follow-up, most CCSs show mild reductions that are often still within normal limits, making it difficult to use a single GLS value as a clinical decision criterion.

Other studies, including the pilot study by Mokshagundam et al. on 78 childhood cancer survivors, suggest that alterations in longitudinal strain may correlate with biomarkers and cardiovascular magnetic resonance (CMR) parameters (extracellular volume, ECV), supporting the idea that strain is a sensitive indicator of subclinical damage when included in a multimodal approach (*p* = 0.05) [25,27,28].

In 25 patients being treated for relapsed AML with CPX-351, Leger et al. showed that, in a population heavily pretreated with anthracyclines, GLS tends to worsen numerically (ΔGLS ≈ +0.9%, therefore less negative), although without reaching statistical significance, while LVEF is significantly reduced in a significant proportion of patients. even in this context, strain adds information compared to ejection fraction (EF) alone, but the extent of the change is relatively small (*p* = 0.03) [29].

Across consensus documents and guidelines, GLS is consistently recognized as a marker of subclinical myocardial vulnerability, yet its interpretation remains context-dependent, influenced by age, body size, vendor variability, treatment intensity, and baseline myocardial reserve. At present, GLS appears more suitable for risk enrichment than for treatment decision-making.

### 5.3. Open Issues in Cardiovascular Magnetic Resonance Cardiotoxicity Assessment

Cardiovascular magnetic resonance (CMR) represents the gold standard for myocardial tissue characterization; however, it is not yet standardized in routine pediatric cardio-oncology practice.

The pilot study by Mokshagundam et al. in paediatric cancer survivors showed that CMR ECV correlates significantly with cumulative anthracycline dose [25]. ECV is also associated with N-terminal-pro-brain natriuretic peptide (NT-proBNP) levels and strain echo parameters (ALS), suggesting that CMR with ECV can identify subclinical diffuse myocardial damage in patients with EF still within normal limits (*p* < 0.001). The authors propose a multimodal surveillance model in which CMR, echo strain and biomarkers are integrated to stratify risk.

An important contribution also comes from the study by Jin et al. on 217 children with ALL undergoing CMR during chemotherapy: in this cohort, all CMR strain parameters (global radial, circumferential and longitudinal strain) were worse than controls, and, in particular, GLS and GRS were independent predictors of adverse clinical events, while the ventricular remodelling index (LVRI) predicted major cardiac events. In this study, a CMR GLS < −18% identified a subgroup with a significantly higher risk of clinical and cardiac events (HR = 5.94, 95% CI 1.23–28.58, *p* = 0.03) [30].

With regard to right ventricular function, Ostler et al. compared echocardiographic measurements [tricuspid annular plane systolic excursion (TAPSE), right ventricle (RV) strain, 3D-right ventricle ejection fraction (RVEF)] and CMR parameters in a cohort of subjects with a history of cardiotoxic exposure, showing good correlation between TAPSE and CMR RV stroke volume (r = 0.392, *p* = 0.003), while RV strain and 3D-RVEF measurements showed more variable performance; CMR remains the gold standard for accurate quantification of RV function, but advanced echocardiography offers a practical window for serial screening [31].

In summary, CMR data in pediatric cardio-oncology delineate two complementary lines of evidence. On one hand, smaller studies confirm CMR as the reference standard for accurate ventricular quantification—particularly for right ventricular assessment—and help identify which echocardiographic parameters are most reliable for serial follow-up. On the other hand, larger cohorts suggest that CMR-derived strain and remodeling indices may detect subclinical cardiotoxicity with potential prognostic relevance and proposed operational cut-offs (such as GLS < −18%). However, due to practical barriers as well as the lack of multicenter validation of CMR-based cut-offs and decision-making algorithms, CMR currently serves more as a phenotyping and adjudication tool than as a population-level screening modality [16].

### 5.4. Open Issues in Cardiac Biomarkers Cardiotoxicity Assessment

Cardiac biomarkers represent a pillar of the surveillance strategy, although paediatric evidence is less established than in adults.

In the study by Mokshagundam et al., NT-proBNP showed a significant correlation with ECV CMR, cumulative dose of anthracyclines, and average longitudinal strain (ALS) on echocardiography, indicating that an increase in BNP may signal both structural remodelling (fibrosis) and subclinical functional damage (*p* = 0.05). Conventional troponin I, on the other hand, was not elevated in that cohort, likely due to limitations in sensitivity and timing [25].

The study by Leger et al. on paediatric patients with AML treated with CPX-351 showed baseline hs-cTnT and NT-proBNP values frequently already altered in a heavily pretreated population, a significant increase in hs-cTnT after the CPX-351 cycle, compared with a stably elevated but not further increased NT-proBNP, no clear correlation between biomarker levels and LVEF at the end of treatment [29]. These results suggest that hs-cTnT may be sensitive to further acute damage, while NT-proBNP reflects an already established state of chronic stress.

In addition to troponin and BNP, “unconventional” biomarkers are being explored. In the pilot study by Mokshagundam et al., suppressor of tumorigenicity receptor 2 (sST2) protein was elevated in a proportion of 66 patients, although without strong correlations with cardiac function in that limited cohort; its potential usefulness requires larger longitudinal studies [25]. Investigations of markers related to the cardiorenal axis, such as α-Klotho (1331.4 ± 735.5 pg/mL vs. 566.43 ± 157.7 pg/mL, *p* < 0.0001) and FGF23 (42.6 ± 18.9 pg/mL vs. 37.4 ± 16.7 pg/mL, *p* = 0.334), have shown alterations compared to healthy controls, with a possible role as indicators of organ damage and overall cardiovascular risk, although in the CCS studied by Rogowicz-Frontczak et al., no significant correlation with echocardiographic EF or SF emerged [32].

Although current studies in pediatric cardiac oncology show variable correlations, the soluble sST2 deserves particular attention. sST2 is a well-known marker of mechanical stress, inflammation, and myocardial fibrosis, which acts by blocking the protective action of inteleukin-33 on the heart. Chronic elevation of sST2 levels may indicate a subclinical response to chemotherapy-induced damage and impending ventricular remodeling, making it a promising candidate for the early identification of high-risk patients before contractile dysfunction occurs.

None of the currently available biomarkers can be recommended as stand-alone screening tools in asymptomatic pediatric patients. Emerging biomarkers (ST2, myeloperoxidase, fibroblast growth factors-23/α-Klotho) promise to contribute to multiparametric risk stratification in the future, especially when integrated with advanced echocardiography and CMR.

## 6. Management and Treatment of Cardiotoxicity in Paediatric Cancer Patients

### 6.1. Primary Prevention During Therapy

Primary prevention is operationalized as early identification of a predefined high-risk subgroup—based on cumulative anthracycline exposure, cardiac irradiation thresholds, relevant comorbidities, and specific modern therapies—and referral to specialized cardio-oncology services for standardized baseline assessment and therapy-tailored surveillance during active treatment. All patients classified as high risk should undergo a standardized baseline cardiovascular assessment, including point-of-care measurements (blood pressure and ECG), evaluation of metabolic syndrome risk, and detailed clinical and family history with lifestyle assessment. For imaging surveillance, the Delphi Consensus recommends transthoracic echocardiography—preferably 3D—with assessment of LVEF and GLS (or 2D LVEF + GLS when 3D is unavailable), while CMR should be considered when repeated TTE examinations are technically suboptimal, if accessible [16].

A tailored primary prevention is currently only possible for anthracyclines and radiotherapy. The only molecule that has shown a cardioprotective effect against anthracyclines is dexrazoxane, which is capable of reducing acute damage without increasing secondary tumours [33,34]. On the contrary, unlike in adults, prolonged infusion or the use of liposomal anthracyclines (CPX-351) has not shown solid evidence in paediatric patients [14,29]. From a radiotherapy perspective, reducing the average dose to the heart to <5–10 Gy substantially reduces the risk of long-term cardiovascular toxicity. Proton therapy could minimise the dose, but paediatric-specific data are still limited [21].

### 6.2. Secondary Prevention

Secondary prevention is based on monitoring and early management of subclinical alterations. The recommended approach is combined monitoring of GLS, RV strain and biomarkers, as reduction/delay of chemotherapy based solely on GLS is not supported by paediatric evidence [14]. With regard to biological therapies, however, longitudinal monitoring must be targeted in each drug class [16].

In the case of TKIs, attention should be focused on controlling hypertension, monitoring QTc, and temporary suspension due to LVEF drop. BRAF/MEK inhibitors can cause left ventricular dysfunction, which is often reversible; this should be monitored serially and multi-parametrically. Anti-CD20 agents are generally safer, although there remains a documented risk of bradyarrhythmias. In the case of mTOR inhibitors, attention should be focused on managing the metabolic profile.

Immunotherapeutic drugs, on the other hand, show a spectrum of immune–inflammatory class side effects. ICI-induced myocarditis should be managed by immediately suspending treatment and starting intravenous methylprednisolone or, in severe cases, immunomodulators such as ATG or abatacept. During CAR-T therapy, the most serious complication remains CRS, in which the use of tocilizumab has been shown to reduce the incidence of cardiogenic shock.

Alongside strategies tailored to each treatment, it is possible to add generic cardioprotective drugs. Adults benefit from angiotensin-converting enzyme (ACE) inhibitors/beta-blockers, but data in children are scarce. The study on paediatric carvedilol did not demonstrate significant prevention. ACE inhibitors show benefits in small studies, but without definitive trials [14]. To date, ACE inhibitors and/or beta-blockers are recommended irrespective of symptoms only in patients with an LVEF < 40% [16]. Innovative approaches such as Ang-(1–7) in animal models have differentiated promising protective pathways [35].

Transplantation is a valid option in refractory cases. The results in patients with anthracycline cardiomyopathy are comparable to those of other cardiomyopathies. Attention should be paid to metabolic comorbidities and residual oncological risk [22].

Given the lack of randomized pediatric trials for ACE inhibitors/beta-blockers, nonpharmacological management remains crucial. Promoting aerobic physical activity should be an essential component of cardiac follow-up. Exercise, if well tolerated, can improve functional capacity, cardiorespiratory fitness, and the management of metabolic risk factors (obesity, dyslipidemia, hypertension), which are often concomitant.

### 6.3. Follow-Up and Survivorship Clinics

These patients typically develop long-term complications after several years. Therefore, several predictive models have been proposed.

Structured models such as the STAR Programme improve adherence and outcomes by integrating cardiologists, paediatric oncologists, nutritionists, physiatrists and psychologists [11].

The CCSS 2025 has developed a predictive model of cardiovascular risk with excellent discrimination (C-statistic 0.78), useful for personalised follow-up. This scoring system assigns points to key risk factors, anthracycline dose (0–3 points), chest radiation dose (0–3 points), age at diagnosis (0–2 points), sex (0–1 point), and body mass index category (0–2 points), with a total score range of 0–11. Scores ≥7 identify survivors at high risk (>25% 30-year cumulative incidence) who may benefit from enhanced cardiovascular surveillance.

## 7. Future Perspectives

A critical gap in evidence persists within pediatric cardio-oncology, a challenge that currently leads to significant variability in clinical practice and contributes to the underdiagnosis and undertreatment of cardiovascular risk factors in this vulnerable patient group. To effectively bridge this divide, there is an urgent and pressing need for international collaborative research. The overarching goal of future efforts is to fundamentally transform pediatric cardio-oncology into a predictive and preventive specialty.

This transformation will largely depend on improving the diagnostic precision and prognostic value of cardiovascular imaging tools. A major unmet need is the validation of conventional echocardiographic parameters of systolic and diastolic dysfunction in large, prospective pediatric cohorts, in order to clarify their true predictive value for clinically meaningful cardiovascular outcomes beyond short-term changes in LVEF or filling patterns. With respect to myocardial deformation imaging, enhancing the clinical utility of strain will require longitudinal studies linking GLS trajectories to hard outcomes, the establishment of age-, sex-, and vendor-specific reference ranges with Z-score-based interpretation, and its integration into multiparametric diagnostic models combining echocardiography, circulating biomarkers, and cardiovascular magnetic resonance (CMR). In parallel, greater access to CMR and the development of standardized, structured reporting protocols are needed to harmonize its use across centers and facilitate comparability of data.

Moreover, future advances in cardio-oncology will need to focus on several additional key directions. Developing more precise, individualized risk stratification models by integrating crucial factors such as genetics, novel biomarkers, and advanced imaging techniques. Defining more effective, evidence-based cardioprotective strategies. Establishing uniform surveillance and follow-up protocols that are proactive, standardized, and ensure a seamless transition for survivors into adult care. These patients typically develop long-term complications after several years. Promoting the integration of lifestyle interventions and metabolic risk factor management as core components of long-term care. Ultimately, these combined efforts aim to ensure that all childhood cancer survivors achieve not only an extended life expectancy, but also a significantly improved cardiovascular quality of life.

## 8. Conclusions

The analysis shows a diagnostic uncertainty, which translates into delayed or inconsistent clinical decisions, particularly in asymptomatic survivors. A critical gap in evidence within pediatric cardio-oncology exists, leading to broad variability in clinical practice and the underdiagnosis of risk factors. It has become clear that conventional parameters, such as EF, are insufficient for early diagnosis, making the adoption of more sensitive techniques—like GLS and CMR with mapping—essential. To overcome this scenario, the specialty must evolve through international collaborative commitment, with the primary goal of becoming a fully predictive and preventive field. Future directions are focused on creating personalized risk stratification models that integrate genetic evaluation, novel biomarkers, and advanced imaging. Concurrently, it is crucial to define evidence-based cardioprotective strategies and establish uniform, proactive surveillance protocols. The ultimate objective is not merely to extend the survival of pediatric oncology patients, but to ensure they achieve a significant and lasting cardiovascular quality of life through holistic care that includes metabolic risk factor management and lifestyle integration. Without this shift, pediatric cardio-oncology risks remaining a reactive discipline rather than evolving into a truly preventive one.

## Figures and Tables

**Table 1:** Pharmacological classes with their respective therapeutic indications and main cardiovascular toxicities.

Treatment Class	Example Drugs	Main Pediatric Malignancies	Estimated % Exposed Today	Estimated % in 5–10 Years	Relevant Cardio-Oncology Notes
**Conventional chemotherapy with anthracyclines**	*Doxorubicin*, *daunorubicin*, *idarubicin*, *epirubicin*, *mitoxantrone*	ALL, AML, HL/NHL lymphomas, sarcomas, neuroblastoma, solid tumors	70–80%	70–80%	Major driver of dilated cardiomyopathy and myocardial hypotrophy; key determinant of late cardiotoxicity
**Alkylating-agent chemotherapy**	*Cyclophosphamide*, *ifosfamide*, *busulfan*, *melphalan*, *procarbazine*, *temozolomide*	Leukemias, lymphomas, sarcomas, neuroblastoma, CNS tumors, high-dose regimens + HSCT	80–90%	80–90%	Myocardial dysfunction; vascular and metabolic toxicity contributing to secondary CV risk
**Liposomal anthracycline formulations**	*CPX-351*	High-risk or relapsed pediatric AML	<1%	1–2%	Potentially reduced cardiotoxicity; limited pediatric evidence
**ABL-targeting TKIs**	*Imatinib*, *dasatinib*, *nilotinib*, *ponatinib*	Pediatric CML, Ph + ALL	1–2%	2–3%	QT prolongation, hypertension, LV dysfunction
**ALK inhibitors**	*Crizotinib*, *lorlatinib*	ALCL ALK+, ALK-mutant neuroblastoma	<1%	1–2%	Bradyarrhythmias, QTc prolongation, possible LV dysfunction
**BRAF/MEK inhibitors**	*Dabrafenib*, *trametinib*, *vemurafenib*, *encorafenib*	BRAF-mutant pLGG, rare pediatric melanoma	0.5–1%	1–2%	Generally mild, reversible LV dysfunction; requires strain monitoring
**Multi-kinase/anti-VEGF TKIs**	*Sunitinib*, *sorafenib*, *pazopanib*, *lenvatinib*, *regorafenib*, *cabozantinib*	r/r sarcomas, thyroid cancers, liver tumors, neuroblastoma	1–2%	2–3%	Hypertension, LV dysfunction, arterial/venous ischemic events
**mTOR inhibitors**	*Everolimus*, *sirolimus*, *temsirolimus*	SEGA, renal tumors, NET	<1%	≈1%	Metabolic toxicity increasing CV risk (dyslipidemia, hypertension, insulin resistance)
**Anti-CD20**	*Rituximab*	Burkitt, pediatric DLBCL, some B-ALL subsets	3–5%	3–5%	Infusion hypotension, rare arrhythmias; usually combined with anthracyclines
**Bispecific antibodies**	*Blinatumomab*, *inotuzumab*	r/r or MRD + B-ALL	2–3%	3–5%	CRS-related cardiac dysfunction, often on anthracycline-injured substrate
**Anti-GD2 antibodies**	*Dinutuximab*, *naxitamab*	High-risk neuroblastoma	2–3%	2–3%	Catecholamine-mediated hemodynamic instability; transient cardiac effects
**CAR-T cell therapies**	*Tisagenlecleucel*, *investigational CD22/BCMA/GD2 CAR-T*	r/r pediatric/YA B-ALL; emerging lymphoma/solid tumor indications	0.5–1%	1–2%	CRS/ICANS-related acute CV toxicity: hypotension, LV dysfunction, arrhythmias
**Immune checkpoint inhibitors**	*Pembrolizumab*, *nivolumab*, *ipilimumab*	r/r cHL, MSI-H/dMMR solid tumors, TMB-high tumors	1–2%	3–5%	Immune-mediated myocarditis (rare but severe), pericarditis, arrhythmias, LV dysfunction

ALCL: anaplastic large-cell lymphoma; ALK: anaplastic lymphoma kinase; ALL: acute lymphoblastic leukemia; AML; acute myeloid leukemia; BCMA: B-cell maturation antigen; BRAF: B-rapidly accelerated fibrosarcoma; CAR-T: chimeric antigen receptors therapy; CD; cluster of differenzation; CML: chronic myeloid leukemia; CRS/ICANS: cytokine release encephalopathy syndrome/immune effector cell-associated neurotoxicity syndrome; CV: cardiovascular; DLBCL: diffuse large B-cell lymphoma; GD2: disialoganglioside; HL/NHL: Hodgkin’s lymphoma/Non-Hodgkin’s lymphoma; HSCT: haematopoietic stem cell transplantation; LV: left ventricle; MEK: mitogen-activated extracellular signal-regulated kinase; MSI-H/dMMR: microsatellite instability-high/deficient mismatch repair; mTOR: mammalian target of Rapamycin; SEGA: subependymal giant cell astrocytoma; TKI: tyrosin kynase inhibitor; TMB: tumour mutational burden; VEGF: vascular endothelial growth factor.

## Data Availability

No new data were created or analyzed in this study. Data sharing is not applicable to this article.
